# Cardioprotective Properties of the Platelet P2Y_12_ Receptor Inhibitor, Cangrelor: Protective in Diabetics and Reliant Upon the Presence of Blood

**DOI:** 10.1007/s10557-015-6609-2

**Published:** 2015-07-16

**Authors:** R. M. Bell, V. Sivaraman, S. P. Kunuthur, M. V. Cohen, J. M. Downey, D. M. Yellon

**Affiliations:** Hatter Cardiovascular Institute, Institute for Cardiovascular Science, University College London, 67 Chenies Mews, London, WC1E 6HX United Kingdom; Department of Physiology and Cell Biology and Medicine, University of South Alabama College of Medicine, Mobile, AL USA

**Keywords:** Cangrelor, P2Y12 inhibitor, Cardioprotection, Ischaemia, Reperfusion injury, Anti-platelet

## Background

Dual antiplatelet therapy represents a cornerstone of the current management of acute coronary syndromes. Combining pharmacological anti-platelet agents, the cyclo-oxygenase (COX) inhibitor aspirin, with a purine P2Y_12_ receptor inhibitor, these pharmacological platelet inhibitors are orally loaded at the time of diagnosis prior to the patient entering the cardiac catheter laboratory. Until recently, the primary physiological and pharmacological focus of these agents has been appropriately directed towards their ability to alter the rheology of the blood; reducing platelet aggregability to attenuate the risk of stent thrombosis. However, there is now an increasing awareness of the pleotropic properties of anti-platelet therapies, particularly of the P2Y_12_ inhibitors, to ameliorate myocardial ischaemia/reperfusion injury.

Recently, it has been demonstrated that administration of P2Y_12_ inhibitors prior to the onset of reperfusion can result in a significant reduction of infarct size. Interestingly, this appears to be a class-effect, with a range of chemically distinct (thienopyridine and non- thienopyridine) P2Y_12_ inhibitors demonstrating the same cardioprotective ability to ameliorating infarct size, provided that an adequate circulating concentration of the inhibitor was present at the moment of reperfusion [1]. The critical difference between thienopyridine and non-thienopyridine P2Y_12_ inhibitors concerns the rate of onset. The first P2Y_12_ inhibitor to gain widespread acceptance in the management of acute coronary syndromes was the thienopyridine clopidogrel. Clopidogrel, like the other widely used thienopyridine, prasugrel, requires a significant period of time to realise P2Y_12_ inhibition by virtue of the need for hepatic P450-mediated conversion of the pro-drug into its active metabolite [2]. The requirement for metabolic conversion is not a problem with the non-thienopyridine P2Y_12_ inhibitors such as Ticagrelor and Cangrelor [2], but the absorption time from the gut—particularly in patients receiving opiate analgesia—will delay the onset of adequate P2Y_12_ inhibition for drugs with an oral route of administration [3]. With a rapid onset of action and the ability to quickly and reliably load the circulation through intravenous bolus and subsequent infusion, Cangrelor is perhaps the ideal antiplatelet therapy for use in the emergency management of ST-segment elevation myocardial infarction, ensuring rapid and effective platelet inhibition at the time of revascularisation and stent deployment. The hitherto unexpected (and unrealised) potential clinical advantage of Cangrelor is the amelioration of ischaemia/ reperfusion injury. Not currently targeted as part of routine clinical management, the excess myocardial cell death resulting from the restoration of blood and oxygen supply can contribute up to 50 % of the final infarct size in experimental and clinical studies [4]. Ischaemia/reperfusion injury therefore represents a clear and currently unmet clinical need—a need that could be met through re-purposing of a therapeutic intervention that is already in widespread clinical practice, for which an intravenous P2Y_12_ inhibitor would be ideally suited.

The mechanism by which platelet P2Y_12_ inhibition mediates its protection remains unclear, but in this communication, we provide further evidence that the protection is dependent upon the presence of blood, and that, unlike other forms of cardioprotection such as ischaemic conditioning [5], we demonstrate for the first time that P2Y_12_ inhibitors can result in significant attenuation of injury of myocardium of animals with diabetes, an important co-morbidity in the cohort of patients presenting with an acute coronary syndrome.

## Methods

All work was conducted in accordance with the Guidelines on the Operation of the Animals (Scientific Procedures) Act 1986, published by The Stationery Office (London, UK), conforming with National Institute of Health Guidelines for the Care and Use of Laboratory Animals. Cangrelor was kindly supplied by the Medicines Company (NJ, USA), and made up into solution in normal saline.

### Ex-Vivo Mouse Heart Perfusion

Male C57BL6 mice (3–4 months of age, 20–30 g weight) were anaesthetized with an intra-peritoneal injection of 60 mg/kg pentobarbitone. The heart was harvested via a para-medial thoracotomy and rapidly transferred to a dissection dish filled with ice cold Krebs–Henseleit buffer and the aorta cannulated with a 21-gauge cannula. The heart was transferred and retrogradely perfused on a Langendorff apparatus, using a modified Krebs–Henseleit buffer (concentrations in mmol/L: NaCl 118.5, NaHCO_3_ 25, glucose 11, KCl 4.7, KH_2_PO_4_ 1.2, MgSO_4_ 1.2, CaCl_2_ 1.8) at 80 mmHg pressure. Each Langendorff perfused mouse heart was randomly assigned to one of the reperfusion protocols, with the reperfusion perfusate fortified with either NaCl vehicle (control) or 20, 200 or 400 nmol/L Cangrelor, for the duration of reperfusion. All hearts are stabilized for 20 min prior to being subjected to 35 min global, normothermic (37.0 ± 0.2 °C) ischaemia and 30 min reperfusion prior to determination of infarct size by triphenyl tetrazolium chloride (TTC) staining as described below.

### In-Vivo Mouse Ischaemia/Reperfusion

C57BL6 male mice (age/weight characteristics as above) were anaesthetized by intraperitoneal injection with a combination of ketamine, xylazine and atropine (0.01 ml/g) to produce final concentrations of 10 mg/ml, 2 mg/ml and 0.06 mg/ml respectively, and the body temperature maintained at 37 °C. The external jugular vein and carotid artery were isolated and cannulated for drug administration and mean arterial blood pressure (MABP) measurement, respectively. A tracheotomy was performed for artificial respiration at 120 strokes/min and 200 μl tidal volume using a rodent Minivent (type 845; Harvard Apparatus, Kent, UK) and supplemental oxygen supplied. A limb lead I electrocardiogram (ECG, LabChart, AD Instruments, UK) was recorded. A left anterior thoracotomy and a chest retractor were used to expose the heart. Ligation of the left anterior descending (LAD) coronary artery was performed using an 8/0 prolene monofilament polypropylene suture. Successful LAD coronary artery occlusion was confirmed by the presence of ST-segment elevation on ECG and a decrease in MABP. Ischaemic conditioning was used as a positive control, comprising of a single cycle of 5 min ischaemia and 5 min reperfusion was effected by temporary LAD ligation. All hearts were subjected to 35 min injurious regional ischaemia, followed by 2 h reperfusion. Ten minutes prior to the onset of reperfusion, the animals were treated with an intravenous 10 min bolus infusion of either vehicle (normal saline) or Cangrelor (60 μg/kg) followed by a continuous infusion of either the vehicle or Cangrelor (6 μg/kg/min) upon the onset and for the duration of reperfusion. At the end of the reperfusion, the heart was isolated, the aortic root was cannulated and infarct size and risk zone determined by TTC and Evans blue staining respectively, as described below.

### In-Vivo Rat Ischaemia/Reperfusion

Male diabetic Goto-Kakizaki rats (3–4 months of age, 250–300 g, University College London, UK) were used in this study. Once anesthetized with sodium pentobarbital (50 mg/kg i.p.), the rats were intubated and ventilated with a Harvard ventilator (70 strokes/min, tidal volume: 8–10 ml/kg). Body temperature was maintained at 37 ± 1 °C by means of a rectal probe thermometer attached to a temperature control system (CMA450). Once the animal was stable, a left lateral thoracotomy was performed to expose the heart and a 6-0 suture placed around the LAD. The suture was tightened using a loop system to create an LAD occlusion and regional ischaemia which was confirmed by ST elevation on ECG and blanching of the myocardium. Following 30 min of ischaemia, the myocardium was reperfused for 2 h. As in the mouse protocol described above, the rats were randomly allocated into vehicle control or Cangrelor treatment groups. Cangrelor was administered as a bolus infusion (60 μg/kg) 10 min before reperfusion, followed by continuous infusion (6 μg/kg/min) for the whole of the reperfusion period. At the end of reperfusion, the heart was excised and the aorta cannulated for identification of risk zone (Evans blue dye) and determination of infarct size by TTC staining as described below.

### Determination of Infarct Size

For in-vivo regional ischaemia experiments, at the end of reperfusion, the heart was excised and aorta cannulated to flush the coronary circulation of blood and the LAD then permanently occluded and the heart then perfused with 0.5 % Evans blue dye to delineate the area at risk (AAR).

Mouse hearts from in-vivo experiments were infused with TTC (prior to Evans blue staining) via the aortic cannula, and then frozen and sliced (6–10 slices perpendicular to long axis). For ex-vivo mouse hearts with global ischaemia the entire left ventricle represents the area at risk, and Evans blue infusion was unnecessary. Rat hearts were first frozen, sliced and then incubated in TTC at 37 °C. TTC stained hearts were then transferred to 10 % neutral buffer formalin for 2 h at room temperature to stabilize the staining. The AAR and infarct size were determined by computerized planimetry performed with the National Institutes of Health (NIH) software Image (Bethesda, MD, USA). The AAR was expressed as a percentage of the left ventricle and the infarct size was expressed as a percentage of the AAR.

### Statistical Analysis

Data are shown as mean ± SEM. Pairwise comparisons were made by Student’s t-test. One-way ANOVA was followed by post-test analysis using the Tukey test for multiple comparisons. Two-way ANOVA was carried out followed by Bonferroni correction to test for significance when performing multiple comparisons between different groups. *P* < 0.05 was considered significant.

## Results

### Cangrelor is Protective In-Vivo, But Not Ex-Vivo

**Fig. 1:**
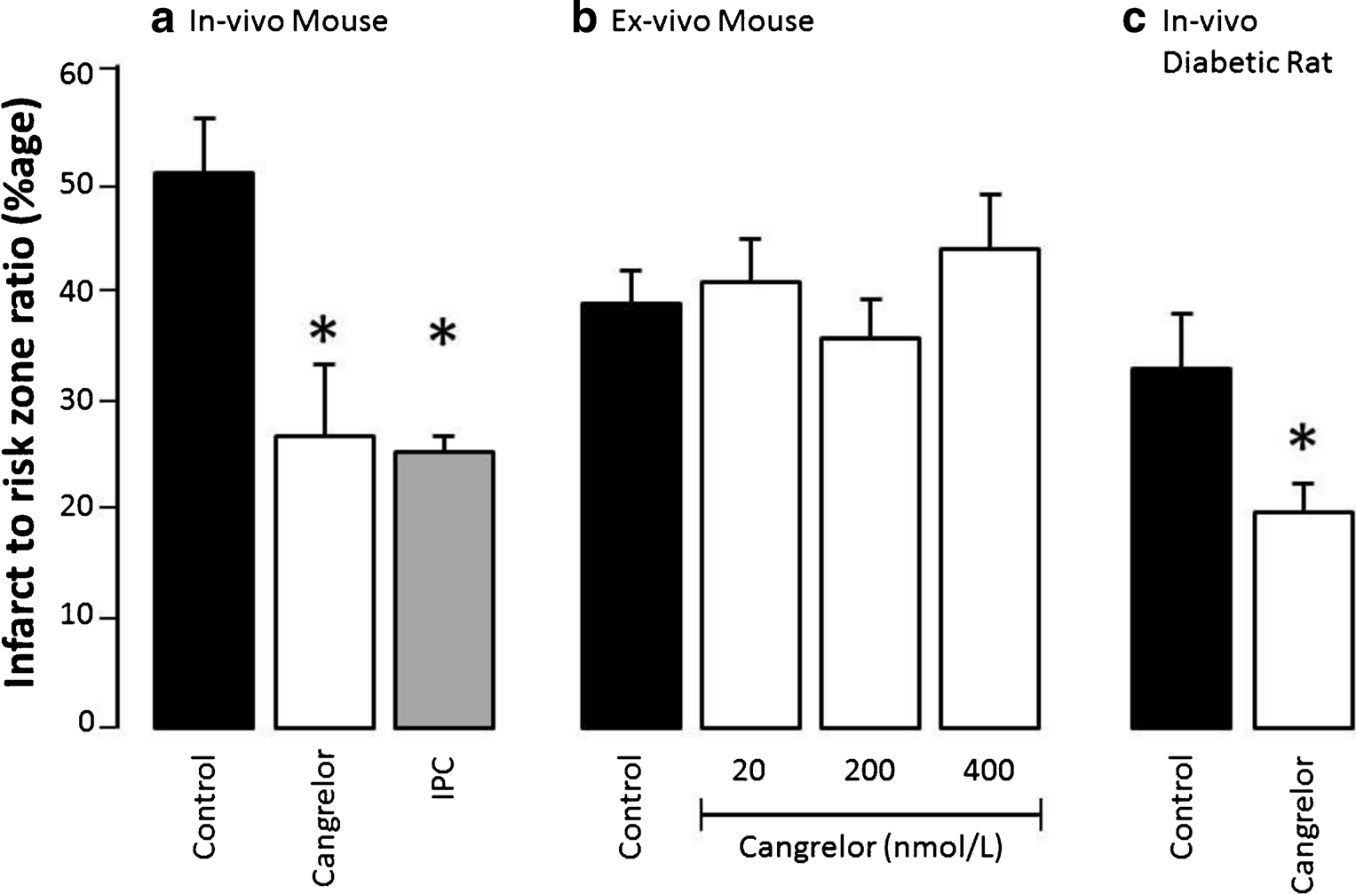
The effect of Cangrelor on infarct size in: **a** mouse in-vivo model; **b** mouse ex-vivo model and **c** the diabetic Goto-Kakizaki in-vivo model. Each bar represents *n* = 4–5 animals per group. For in-vivo experiments, Cangrelor was administered as a single 60 mg/kg bolus 10 min prior to the onset of reperfusion, followed by a 6 mg/kg/min infusion for the whole reperfusion period; *p < 0.05 treatment versus respective saline vehicle control group. For the ex-vivo experiment, the Cangrelor dosing regime is as indicated, with the drug present throughout the reperfusion period. IPC = Ischaemic Preconditioning (1× cycle of 5 min regional ischaemia, 5 min reperfusion)

To determine whether Cangrelor is protective against injurious ischaemia/reperfusion in mice, we studied Cangrelor use in both the ex-vivo and in-vivo setting. As previously demonstrated in a number of different animal models [1, 6, 7], we found that Cangrelor was significantly protective in the murine heart, in-vivo, when administered at the time of reperfusion. Cangrelor attenuated the final infarct size equivalent to that seen following ischaemic preconditioning (52 ± 5 % in control hearts versus 28 ± 6 % with cangrelor and 25 ± 1 % for ischaemic preconditioning, *p* < 0.05, Fig. 1a). This protection was dependent upon the presence of blood, as protection observed in the ex-vivo, crystalloid-perfused setting was not reciprocated. To confirm this effect we further undertook a dose response curve. A cangrelor infusion of 6 μg/kg/min is equivalent to a final blood concentration of 200 nmol/L, but we found no evidence of protection in the isolated heart over a dose-response range of 20–400 nmol/L (39 ± 3 %, 41 ± 3 %, 36 ± 3 % and 44 ± 5 % for control, 20, 200 and 400 nmol/L Cangrelor respectively, *p* = 0.5642, Fig. 1b). Cangrelor was also not protective in crystalloid-perfused rabbit hearts [1], but only a single dose was studied. Here we extend that observation to a second species and a wide range of concentrations.

### Cangrelor is Protective in the Diabetic Myocardium

We have previously demonstrated that the threshold for ischaemic conditioning is significantly elevated in diabetic myocardium [5], requiring a more robust ischaemic conditioning stimulus in order to overcome the nascent resistance to cardioprotective signalling that results from chronic exposure to hyperglycaemia and altered insulin resistance [5]. To determine whether Cangrelor would still result in cardioprotection under these circumstances, we administered Cangrelor at the time of reperfusion to Goto-Kakizaki rats, a lean type 2 diabetic rat derived from the Wistar rat strain [8]. At the drug infusion rates used, which is relevant to human clinical dosing, Cangrelor resulted in a significant attenuation of infarct size compared to the vehicle-treated size (20 ± 2 %) compared to vehicle-treated control diabetic hearts (33 ± 6 %, *p* < 0.05, Fig. 1c). There was no significant difference in the peri-procedural blood glucose level between groups (26.4 versus 22.9 mmol/L in control and Cangrelor groups respectively).

## Discussion

Here we demonstrate that cangrelor is not protective in an ex-vivo, non-blood perfused murine model of ischaemia/reperfusion injury, but is strongly protective in-vivo. This is the first demonstration of the cardioprotective effect of a P2Y_12_ inhibitor in mouse—indicating that it would be possible to tease out the mechanism of this protection using transgenic/knockout models. Given the apparent dependence upon the presence of blood, it would seem logical to propose that the cardioprotective effect of cangrelor is dependent on platelets and likely the platelet P2Y_12_ receptor, an argument given additional weight by previous observations that chemically distinct P2Y_12_ inhibitors such as clopidogrel have similar cardioprotective properties [1]. The protection against infarction does not appear to be the direct result of blocked aggregation as a number of signal transduction inhibitors blocked cangrelor’s protection but did not restore the ability of the platelets to aggregate [1]. We would therefore propose that future work into this highly desirable “side effect” of P2Y_12_ inhibitors—namely the pleotropic attenuation of ischaemia/reperfusion injury, as manifest by a significant reduction of infarct size—should seek to concentrate on identifying cangrelor’s target in the blood (presumably platelet activation) and that target’s role in the evolution of myocardial injury.

In this communication, we also demonstrate that, unlike other forms of cardioprotection such as ischaemic conditioning, cangrelor-mediated protection can be seen in diabetic myocardium. That this is seen when administering cangrelor at a clinically relevant, anti-platelet dose, is of great encouragement: in the face of a predicted impending explosion in the prevalence of diabetes within the general population, the management of ischaemic heart disease is set to become ever more challenging, and continuation of the recent historical trend of improving cardiovascular outcomes from coronary artery disease is likely to become ever more difficult. Our finding indicates that current therapy with a P2Y_12_ blocker has the potential to exert an anti-infarct effect in the diabetic population undergoing PCI provided timely treatment is administered. An understanding of the fundamental similarities and differences between the protective mechanisms of classical cardioprotective strategies, such as ischaemic conditioning, and the novel cardioprotection observed following cangrelor administration should help to elucidate additional pharmacological targets to further improve patient outcomes, particularly in those patients with co-morbidities such as diabetes.
